# Supplementary material for: Pre-saccadic suppression and compensatory cortical distortions support predictive visual remapping
Source: bioRxiv. 2026 Oct 26:2026.10.20.760565. Preprint. [Version 1] doi: 10.64898/2026.10.20.760565 (PMC13733681; doi:10.64898/2026.10.20.760565)
Supplement: Supplementary file 1 [file media-1.pdf]

Monkey M

	Number of neurons involved in the analysis	Number of neurons that exhibit remapping	Ratio of remapping neurons
Superficial layer	53	35	66.0%
Input layer	79	58	73.4%
Deep layer	46	29	63.0%
Total counts	178	122	68.5%

Monkey D

	Number of neurons involved in the analysis	Number of neurons that exhibit remapping	Ratio of remapping neurons
Superficial layer	91	37	40.7%
Input layer	72	34	47.2%
Deep layer	36	11	30.6%
Total counts	199	82	41.2%

Both monkeys

	Number of neurons involved in the analysis	Number of neurons that exhibit remapping	Ratio of remapping neurons
Superficial layer	144	72	50.0%
Input layer	151	92	61.0%
Deep layer	82	40	48.8%
Total counts	377	204	54.1%

Table S1. Laminar distribution of analyzed V2 neurons

Number of total neurons analyzed (restricted to single units with baseline firing rates greater than 3 Hz) and the corresponding number and proportion of neurons exhibiting significant forward RF remapping (see Methods) across cortical layers.

Figure S1

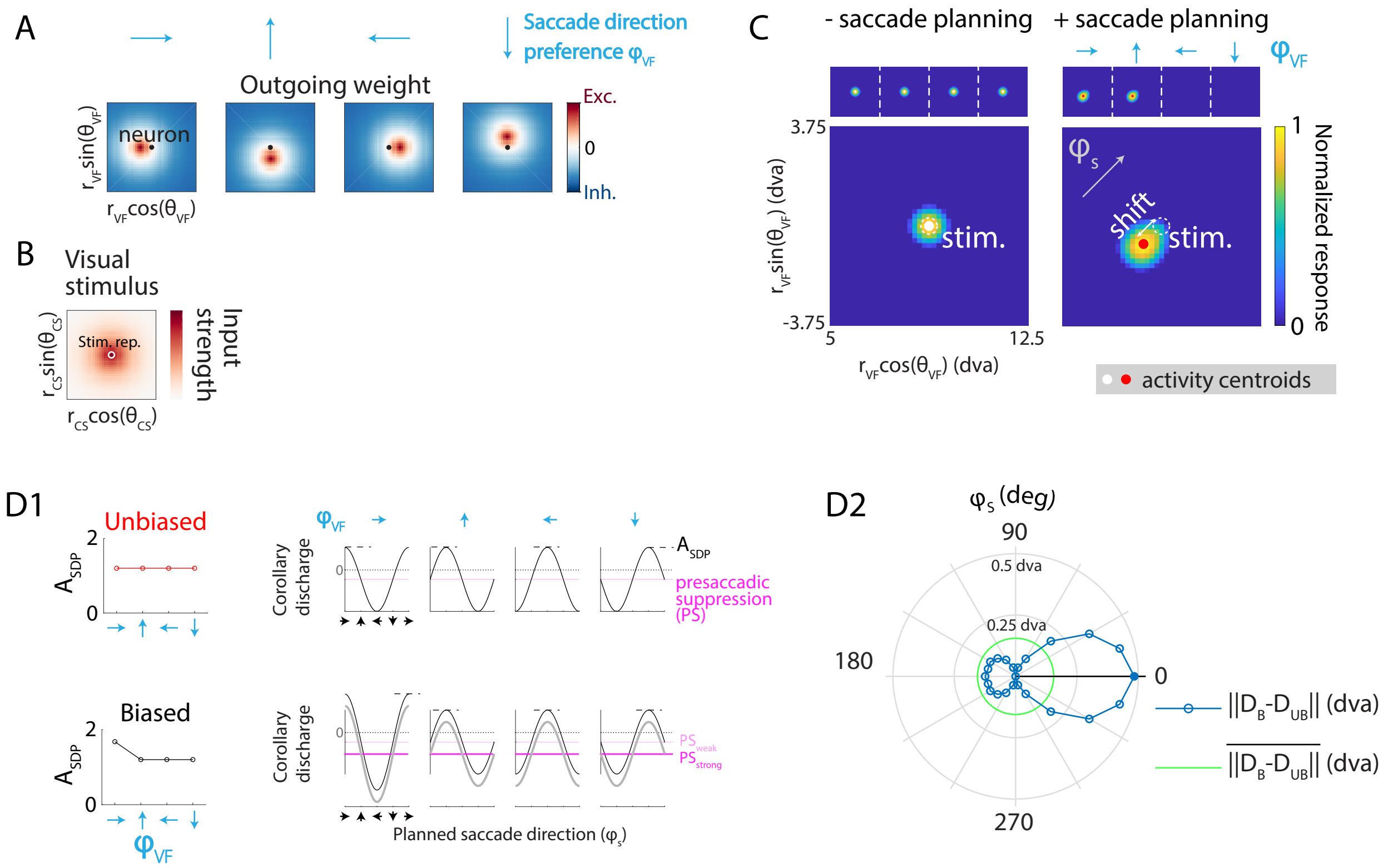

Figure S1. Network model of receptive field (RF) remapping under uniform cortical transformation.

(**A**) Outgoing weights from neurons with same spatial RFs (black dots) but four different SDPs (blue arrows), as a function of the RFs of postsynaptic neurons. (**B**) Amplitude of inputs caused by visual stimulus as a function of neurons' cortical locations. White circle: cortical representation of visual stimulus. (**C**) Steady-state population activity (by RF location) in response to a visual stimulus presented without (left) and with (right) saccade planning. Top row: activity grouped by saccade direction preferences (SDPs). Bottom row: activity averaged across SDPs. White dashed circles: location of the visual stimulus. Grey arrow: planned saccade direction (φ_S). (**D**) Remapping with biased SDP amplitude. **D1**: Left: Unbiased and biased SDP amplitude (A_{SDP}) as a function of SDP (φ_{VF}). Right: Corresponding corollary discharge amplitude for neurons with four different SDPs, as a function of planned saccade direction. **D2**: Remapping error induced by bias in the strength of saccade direction selectivity as a function of planned saccade direction. Black line: direction of SDP along which the bias is introduced. Green circle: mean error across tested saccadic directions (φ_S). Filled circle: maximum error across φ_S. $PS = 2.8$, $A_{SDP} = 1.68$ for $\varphi_{VF,i} = 0°$ under the biased condition and 1.2 otherwise.

Figure S2

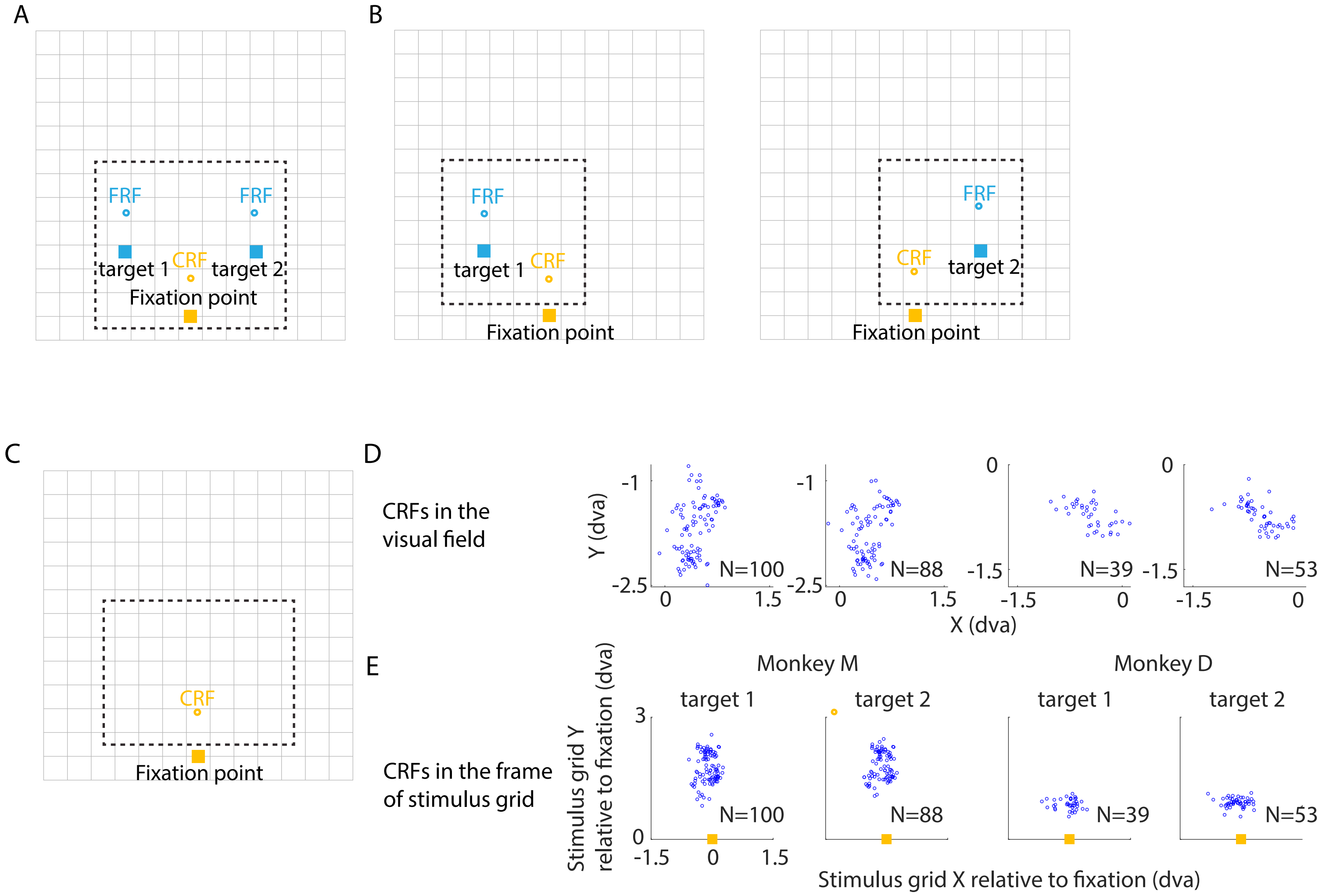

Figure S2. Neuronal responses and RFs under a cued saccade task

(**A**) The area of stimulus locations used to compute the responses under various saccade directions and characterize pre-saccadic suppression. (**B**) Visual regions of interest for RF searching during saccade planning for various saccade directions. (**C**) Visual region of interest for RF searching during the pre-planning period. (**D**) Distribution of current RFs (CRFs) for recorded neurons exhibiting forward remapping under various saccade directions for each monkey. (**E**) Same as (D) but shown in the reference frame of the stimulus grid, where the coordinates of fixation point and saccade targets were fixed across sessions.

Figure S3

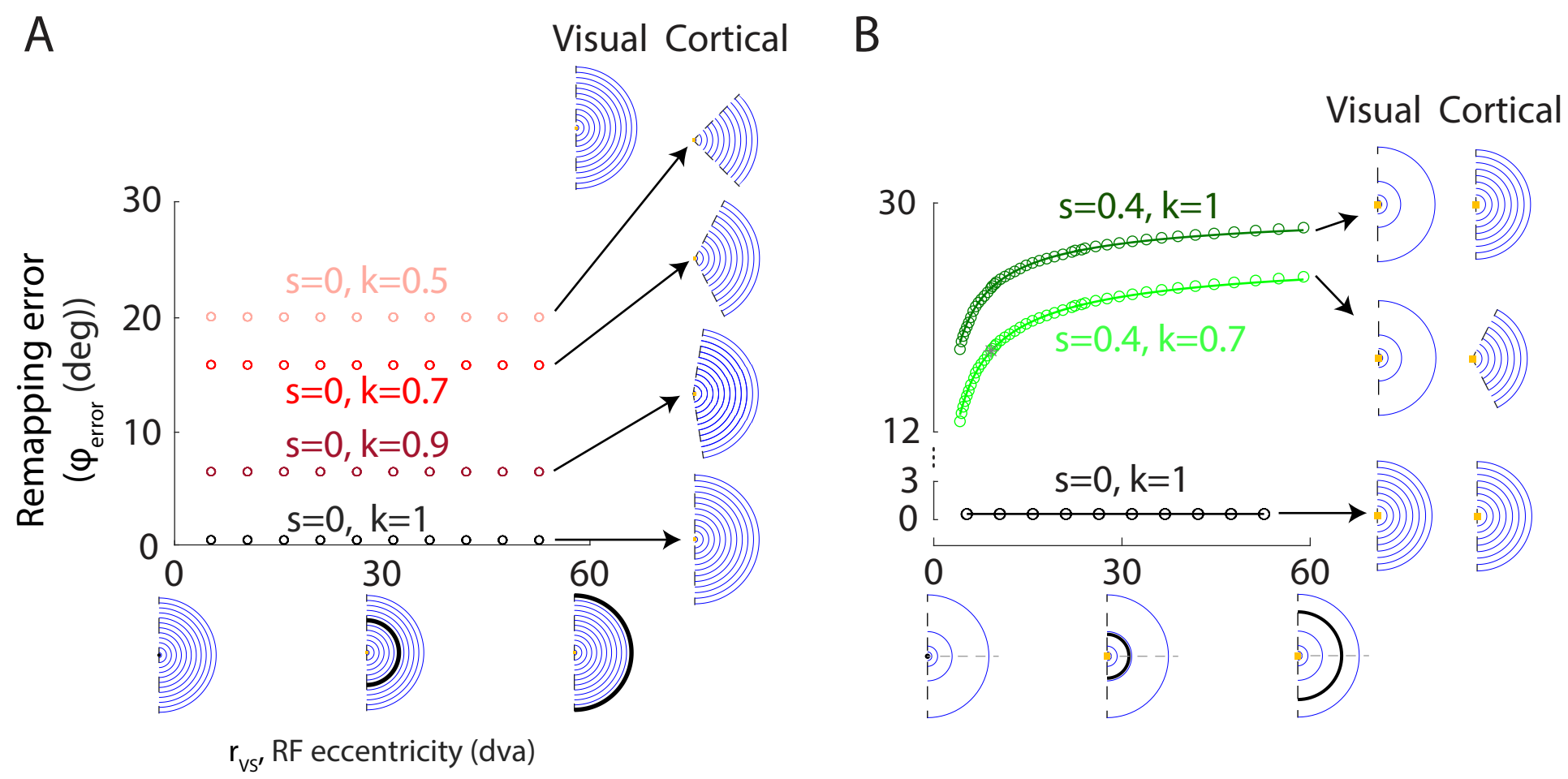

Figure S3. Remapping error under polar angle contraction with eccentricity-independent cortical magnification

(A) Same as Fig. 4D but under conditions of eccentricity-independent cortical magnification (CM) ($s = 0$) across various extents of polar angle contraction (k). The remapping error is independent of CRF eccentricity. (B) Light green: same as Fig. 4D but under conditions of both eccentricity-dependent CM ($s = 0.4$) and polar angle contraction ($k = 0.7$). Black: uniform transformation as shown in Fig. 4D. Dark green: eccentricity-dependent cortical magnification (CM) only as shown in Fig. 4D. Grey asterisk: transformations considered in Fig. 4B, C. Curves: fits using general power functions (see Methods).

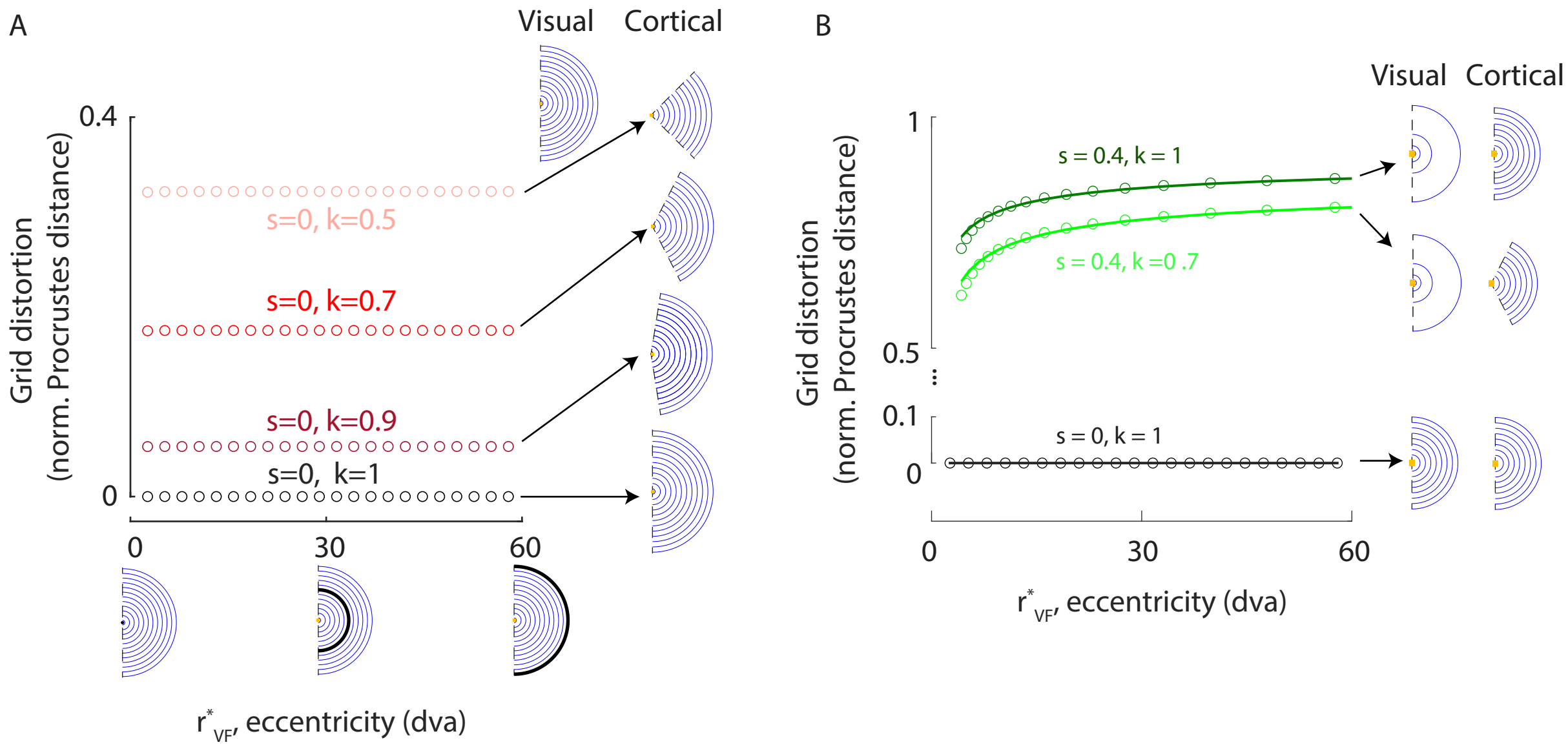

Figure S4. Grid distortion under polar angle contraction with eccentricity-independent cortical magnification

(A) Same as Fig. 6B but under conditions of eccentricity-independent CM ($s = 0$) across various extents of polar angle contraction (k). The grid distortion is independent of the eccentricity of network location. (B) Light green: same as Fig. 6B but under conditions of both eccentricity-dependent CM ($s = 0.4$) and polar angle contraction ($\mathrm{k} = 0.7$). Black: uniform transformation as shown in Fig. 6B. Dark green: eccentricity-dependent cortical magnification (CM) only as shown in Fig.6B.

Figure S5

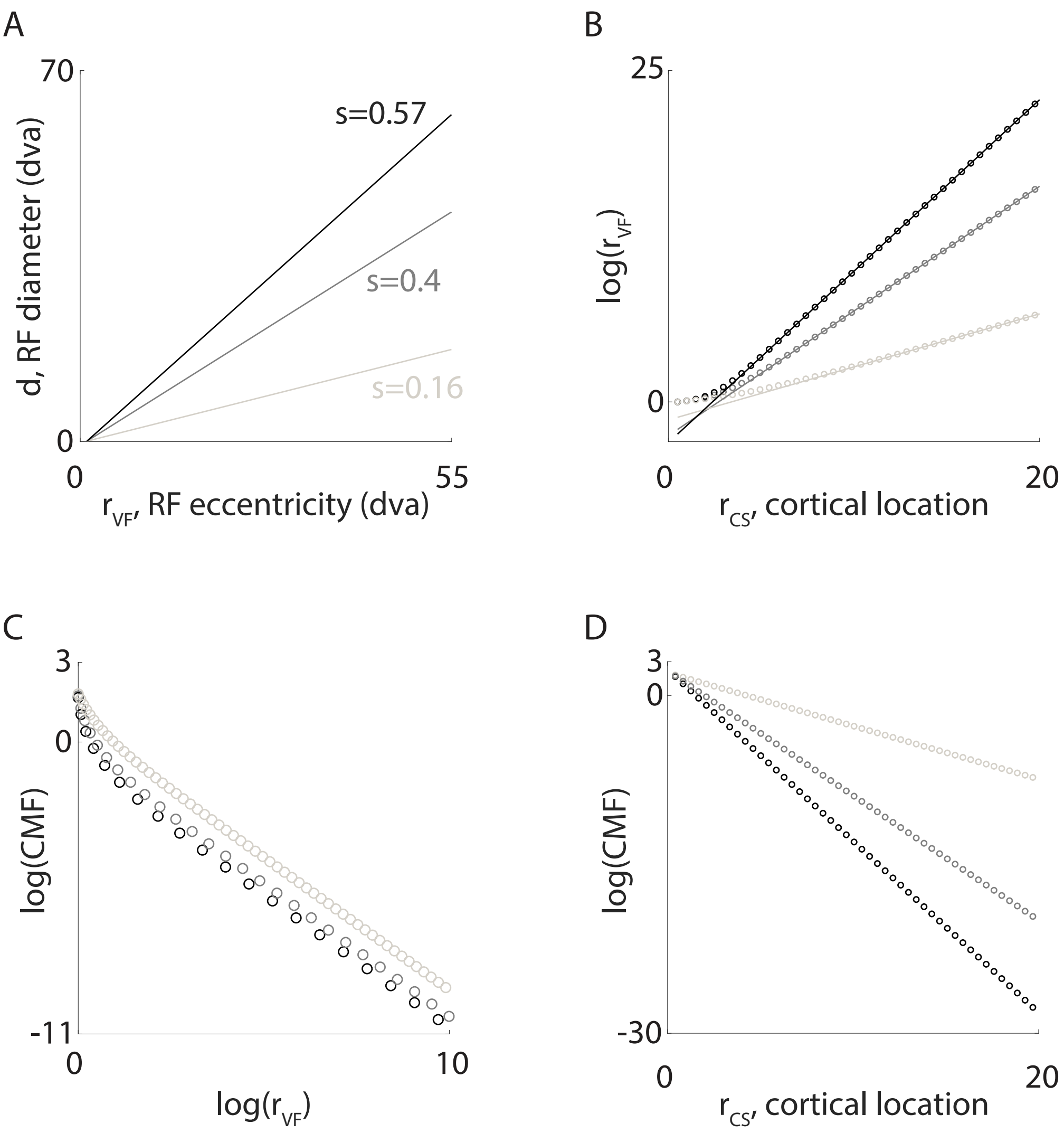

Figure S5. Constructing eccentricity-dependent cortical magnification

(**A**) Linear relationships between RF diameter (d) and RF eccentricity (r_{VF}) explored in the study. (**B**) Corresponding $log(r_{VF})$ as a function of cortical location (r_{CS}), constructed using the linear relationship shown in (A). Circles: constructed data. Lines: theoretical linear relationships (Eq. 13-14). (**C**) Corresponding CM factor (CMF), approximated by forward difference methods based on (**B**). (**D**) Same as (C) but as a function of r_{CS}. $D_c = 0.5$, $d_1 = 0.1$, $\mathrm{r_{VF,1}} = 1$.
